# Investigatıon of Antioxıdative, Cytotoxic, Membrane-Damaging and Membrane-Protective Effects of The Essentıal Oil of *Origanum majorana *and its Oxygenated Monoterpene Component Linalool in Human-Derived Hep G2 Cell Line

**Published:** 2017

**Authors:** Ayse Erdogan, Aysun Ozkan

**Affiliations:** a *Department of Biology, Faculty of Science, University of Akdeniz, 07058, Antalya, Turkey.*

## Introduction

In all aerobic organisms, including human beings, production of reactive oxygen species (ROS) is balanced by antioxidant defense system. A serious imbalance between the production of ROS and antioxidant defense system is responsible for oxidative stress. Thus, ROS plays an important role in the etiology of many diseases and aging. Antioxidant defense system which prevents oxidative damages of ROS consist of flavanoids, carotenoids, phenolic compounds, vitamins, and antioxidant enzymes (1). Although there are some synthetic antioxidants such as butylated hydroxytoluene (BHT) and butylated hydroxyanisole (BHA) which is commonly used in processed food, it has been reported that these compounds have some side effects (2). Recently, there has been increasing interest in finding plants with high antioxidant capacities since they can protect human body from free radicals and retard the progression of many chronic diseases (3-5).

Various medicinal properties have been ascribed to natural herbs. Medicinal plants constitute the main source of new pharmaceuticals and healthcare products. Plant products are also known to possess potential for food preservation.

Sweet marjoram [*Origanum majorana *L. syn. *Majorana hortensis* Moench], a member of the Lamiaceae family, is of great economic and industrial importance (6). Lamiaceae consists of more than 150 species occurring mainly in the Mediterranean area and are used as herbal teas for their (folk) medicinal properties. *Origanum *is represented in Turkey by 22 species or 32 taxa, 21 being endemic to Turkey. Out of 52 known taxa of *Origanum*, 60% are recorded to grow in Turkey (7). Lamiaceae plants were widely studied as natural antioxidant sources because of their high contents of polyphenols (8). 

*O. majorana* is one of the most important aromatic plants that contain major antioxidants such as flavonoids and triterpenoids (9). Phenolic acids and flavonoids have been reported to play a role in the prevention of human pathologies (10). Food and Drug Administration regard *O. majorana *to be generally safe.

Genus *Origanum* is used as anti-diabetic, carminative, tonic, digestive, stimulant, expectorant, menstrual regulator, diuretic, and for respiratory problems such as asthma. Aqueous and ethanolic extracts from marjoram played a role in preventing carcinogenesis and oncogenic mutations (11). These extracts caused alterations in methylguanine-DNA methyltransferase activity which is highly expressed in human cancers. *O. majorana*’s crude drug extracts exerted a low cytotoxicity on five human liver-cancer cell lines studied with an average of 39.1% (12). Commercial *O. majorana* oil is used as a spice and condiment. The oil is used in perfumery for its spicy herbaceous notes (13) and as fungicides or insecticides in pharmaceutical and industrial products (14).

Linalool is monoterpene compound reported to be major volatile components of the essential oils of several aromatic species. Linalool (3,7-dimethyl-1,6-octadien-3-ol) is one of the commonly used chemicals in cosmetic products and the fragrance and perfume industry. Safety evaluation studies revealed that linalool is not irritating, phototoxic or sensitizing but has a low order of acute toxicity (15).

Hepatoma G2 cells (Hep G2) are considered a good model to study *in vitro* xenobiotic metabolism and toxicity to the liver, since they retain many of the specialized functions which characterize normal human hepatocytes (16). Also, Hepatoma G2 cells are a valuable model to study hepatocellular carcinoma and the liver, where drugs are metabolized (17).

In another experiment, we studied phenolic terpenoid carvacrol’s (main component of *O. majorana *essential oil) cytotoxicity, cytoprotective and membrane protective effects on Hep G2 cells (18). Therefore, we used linalool (second main component of *O. majorana* essential oil) for this experiment.

The aim of our work was to compare cytotoxic and membrane-damaging effects of* O. majorana* essential oil and its oxygenated monoterpene component linalool on the human hepatocellular carcinoma cell line Hep G2 and to investigate their possible protective (antioxidant) effects against H_2_O_2_ induced membrane damage.

## Experimental

*Collection of plant material*

*O. majorana *was collected from Mahmutseydi Village, Alanya (730 m) in Turkey (Voucher no: TR 102 AL). The taxonomic identification of plant materials were confirmed by a plant taxonomist, Prof. Dr. **R. Süleyman Göktürk**, in Department of Biology, Akdeniz University, Antalya, Turkey.

*Isolation of the essential oil*

The air-dried aerial parts of plants (100 g) were powdered and subjected to hydrodistillation for 3 h using the Clevenger-type apparatus (ILDAM Ltd., Ankara, Turkey) at Molecular Biology Department in Biology in Akdeniz University. The obtained essential oil was dried over anhydrous sodium sulphate and after filtration, stored at +4°C until tested and analysed. Linalool (purity 97%) was purchased from Aldrich.

*Gas chromatography mass spectrometry (GC–MS)*

The composition of the volatile constituents was established by GC-MS/quadruple detector analyses using a Shimadzu QP 5050 system, equipped with a chemically bonded fused silica column Cp WAX 52 CB (50 m × 0.32 mm internal diameter, 1.2 m film thickness). GC analysis was employed under the following conditions: Injector temperature was 240°C. Detector temperature was 250 °C. The temperature program for the Cp WAX 52 CB column was from 60 °C to 220 °C at a rate of 2 °C/min and than held at 220 °C for 20 min. Helium was used as a carrier gas at a flow 10 psi and injection volume of each sample was 1 mL, split ratio (1:20). The MS conditions were: ionization energy, 70 eV; mass range, 40–400 amu; scan mode, EI. The percentage composition was computed from the GC peak areas according to the 100% method without using any correction factors. The identification of the components was based on the comparison between their mass spectra and those of Wiley and Nist, Tutore Libraries. 

The components were identified based on the comparison between their relative retention time as wells as mass spectra and those of standards and literature data (19, 20). The results were also confirmed by the comparison between the compounds elution order and their relative retention indices on non-polar phases reported in the literature (19, 20). All experiments were carried out at least in triplicate. Theresults were reported as mean values.

*Antioxidant Activity*

*DPPH assay*

The hydrogen atoms or electrons donation ability of the corresponding oils and some pure compounds were measured from the bleaching of purple coloured methanol solution of 2,2-diphenyl-1-picrylhydrazyl (DPPH). This spectrophotometric assay uses stable radical DPPH as a reagent (21, 22). Various concentrations (50 µL) of the essential oil in methanol were added to 5 mL of a 0.004% (w/v) methanol solution of DPPH. After a 30 min incubation period at room temperature the absorbance was read against a blank at 517 nm. Inhibition free radical DPPH in percent (I%) was calculated in following way: 

I % = (A _control_ - A_ sample_/A_control_) x100

Where A_blank_ is the absorbance of the control reaction (containing all reagents except the test compound), and A_sample_ is the absorbance of the test compound. Essential oil and positive controls concentrations providing 50% inhibition (EC_50_) were calculated form the graphs. For the calculation of these values, Microsoft Excel software was used. Tests were carried out in triplicate.

*-carotene–linoleic acid assay*

In this assay antioxidant capacity is determined by measuring the inhibition of the volatile organic compounds and the conjugated diene hydroperoxides arising from linoleic acid oxidation (23). A stock solution of -carotene–linoleic acid mixture was prepared as following: 0.5 mg -carotene was dissolved in 1 mL of chloroform (HPLC grade), 25 μL linoleic acid and 200 mg Tween 40 was added. Chloroform was completely evaporated using a vacuum evaporator. Then 100 mL distilled water saturated with oxygen (30 min, 100 mL/min) was added with a vigorous shaking.

This reaction mixture (2500 μL) was dispersed to test tubes and 350 μL portions of the essential oil prepared at 2 g/L concentrations added and also the emulsion system was incubated up to 24 h at room temperature. The same procedure was repeated with synthetic antioxidant, butylated hydroxytoluene (BHT), ascorbic acid and -tocopherol as positive controls, and a blank. After this incubation period, the absorbance of the mixtures was measured at 490 nm. Antioxidative capacity of the essential oil was compared with those BHT, ascorbic acid, -tocopherol and blank. Tests were carried out in triplicate.

*Cell lines and cell culture*

The hepatoma G2 cells (Hep G2) line (purchased from American Type Culture Collection) was used in this study. Cells were grown in Minimum Essential Medium (MEM) supplemented with 10% (v/v) fetal bovine serum, 1% (v/v) antibiotic-antimycotic solution in a humidified atmosphere containing 5% CO_2_ at 37°C. For subculturing, cells were harvested after trypsin/ethylenediaminetetraacetic acid treatment at 37°C. Cells were used when monolayer confluence had reached 75%.

*Cytotoxicity assay*

The cells were seeded into 96-well microplates (1×10^4^ cells well^−1^) for 24 h and then treated with different concentrations of the essential oil and linalool (5-500 μg/mL) for 24, 48 and 72 h. Cytotoxicity of the essential oil and linalool was assayed by CellTiter-Blue® Cell Viability Assay. The assay is based on the ability of living cells to convert a redox dye (resazurin) into a fluorescent end product (resofurin). Nonviable cells rapidly lose metabolic capacity and thus do not generate a fluorescent signal (24). Following cellular reduction, fluorescence is recorded at 560 nm excitation/590 nm emissions. The data were expressed as average values obtained from eight wells for each concentration. The IC_50 _value was calculated from equation of graph. H_2_O_2_ cytotoxicity on cancer cells was measured in the same way. For measuring antioxidant effect of the essential oil and its oxygenated monoterpene component linalool against H_2_O_2_ cytotoxicity, different concentrations of the cells were preincubated (IC_10_, IC_20_ and IC_30_) for 1 h, before H_2_O_2 _treatment (IC_10_, IC_50_ and IC_70_) for 24 hour. Essential oil and its oxygenated monoterpene component linalool were dissolved in 0.5% (v/v) dimethyl sulphoxide (DMSO).

*Determination of malondialdehyde level*

Malondialdehyde (MDA) levels were determined after Hep G2 cells were exposed to different concentration of essential oil and its oxygenated monoterpene component linalool for 24 h (IC_10_, IC_50_ and IC_70_). For measuring membrane protective effects of the essential oil and linalool against H_2_O_2_, the cells were preincubated with different concentrations (IC_10_, IC_20_ and IC_30_) of essential oil and linalool (IC_10_, IC_20_ and IC_30_) for 1 h, before H_2_O_2_ treatment (IC_10_, IC_50_ and IC_70_) for 24 h. Essential oil and its oxygenated monoterpene component linalool were dissolved in 0.5% (v/v) DMSO.

Hep G2 cells were plated at a density 15×10^4^ cell/100 mm dishes. The cells were scraped off culture plates with culture medium and were centrifuged 600×g for 10 min. The cell pellets were washed with PBS and then sonicated (3×15 sec) in 50 mM potassium phosphate, pH 7.2, containing 1 mM PMSF and 1 μg/mL of leupeptin and centrifuged at 150,000×g for 45 min. The supernatant was used for the determination of malondialdehyde level.

Malondialdehyde levels in Hep G2 cells were assayed as described in the previous method (25). This fluorometric method for measuring thiobarbituric acid-reactive substances (TBARS) in supernatant is based on the reaction between malondialdehyde and thiobarbituric acid. The product of this reaction was extracted into butanol and measured at 525 nm (excitation) and 547 nm (emission) spectrofluorometrically.

Protein was determined by the Bradford method (26) with bovine serum as a standard.

*Data analysis*

The results of the replicates were reported as mean ± standard error. Comparison of treatments against controls was made using a one-way ANOVA; the significance level chosen for all statistical analysis was *p *< 0.05. 

## Results and Discussion

*Chemical composition of the essential oil*

GC/MS analysis of the plant essential oil led to the identification and quantification of 5 components, which accounted for 99.9% of the total oil (Table 1). The major components of the oil were carvacrol (52.5%) and linalool (45.4%). The essential oil consisted mainly of oxygenated monoterpenes (98.2%), whereas monoterpene hydrocarbons were weakly represented **(**1.7%). A portion (0.1%) of the total composition was not identified. Terpinen-4-ol was found as a main component in* Origanum majorana* essential oil which was from Kalocsa, Hungary. Linalool was also found as a component (12.1%) but carvacrol was not found (27).

**Table 1:** Chemical composition of the essential oil from *O. majorana*

**Components**	**RT**	**Composition (%)**
γ-Terpinene	17.4	0.9
Cymene	18.4	0.8
Thymol	65.8	0.3
Carvacrol	67.3	52.5
Linalool	39.3	45.4
Total		99.9

%, relative compound percentage.

RT, retention time (min).

As far as we know, there are many researchers on the investigation of chemical composition of the oils isolated from *Origanum* species (13, 28, 29). It was postulated that the oil exists in two forms. One with terpinen-4-ol and sabinene hydrate as major components (30) and the other with thymol and /or carvacrol (28, 30, 31) as predominant compounds. The marjoram essential oil has shown that the volatile aroma composition varies according to geographic differences and by the climatic features such as temperature (32) thereby altering the biological activities studied (33). 

*The antioxidant activity*

Radical-scavenging activity of the essential oil from aerial parts of *O. majorana *was evaluated by the 2,2-diphenyl-1-picrylhydrazyl (DPPH) radical assay. The concentration that led to 50% inhibition (EC_50_) is 170 μg/mL. Essential oil’s radical-scavenging activity was found 9.2, 52.96 and 23.45 times lower than BHT, ascorbic acid and α-tocopherol respectively (Table 2). 4-terpineol (29.97%), gamma-terpinene (15.40%), trans-sabinene hydrate (10.93), alpha-terpinene (6.86%), 3-cycolohexene-I-1 methanal, a, a4-trimethyl-,(S)-(CAS) (6.54%), and sabinene (3.91%) were found as main constituents of *Origanum majorana* collected from local market of Cairo, Egypt, and its EC_50_ concentration was found to be 58.67 mg/mL (34).

**Table 2:** Antiradical activities and linoleic acid oxidation inhibitions of *O. majorana* essential oil and positive controls.

**Samples**	**DPPH assay (EC**_50_ **, µg/mL)**	**β-Carotene/linoleic acid assay (I%)**
*O. majorana*	170 + 0.3	40.0 + 0.2
BHT	18.5 + 0.2	94.8 + 0.1
Ascorbic acid	3.2 + 0.4	92.0 + 0.3
α-Tocopherol	7.2 + 0.6	96.4 + 0.5

BHT, butylated hydroxytoluene; DPPH, 2,2-diphenyl-1-picrylhydrazyl; I%, inhibition in percentage.

Moreover, compared to other *Origanum* species, the DPPH radical scavenging activity of essential oil from* O. majorana* was found higher than *O. vulgare* ssp. v*ulgare* which consisted of caryophyllene (14.4%) and spathulenol (11.6%) the main constituents, followed by germacrene-D (8.1%) and α-terpineol (7.5%). but lower than *O. onites *which had linalool (50.53%), carvacrol (24.52%) and thymol (15.66%) as three major constituents (18, 35).

The potential of the essential oil to inhibit lipid peroxidation was evaluated using the -carotene/linoleic acid bleaching test. The essential oil showed 40% inhibition. The essential oil’s linoleic acid oxidation inhibition capacity was found 2.37, 2.3 and 2.41 times lower than BHT, ascorbic acid and α-tocopherol respectively (Table 2). *O. vulgare* ssp. *vulgare* was more effective than *O. majorana* in linoleic acid oxidation (35). Inhibition of linoleic acid oxidation capacity of *O. majorana* was equal to *O. onites* capacity (18). Linalool’s antioxidant activity was studied in the previous research. Linalool’s EC_50_ value was calculated as 16.4 μg/mL in DPPH radical assay and linalool showed antioxidant activity in the -carotene/linoleic acid bleaching test (36).

*The antioxidant effects of the essential oil and linalool on Hep G2 cells*

**Figure 1:**
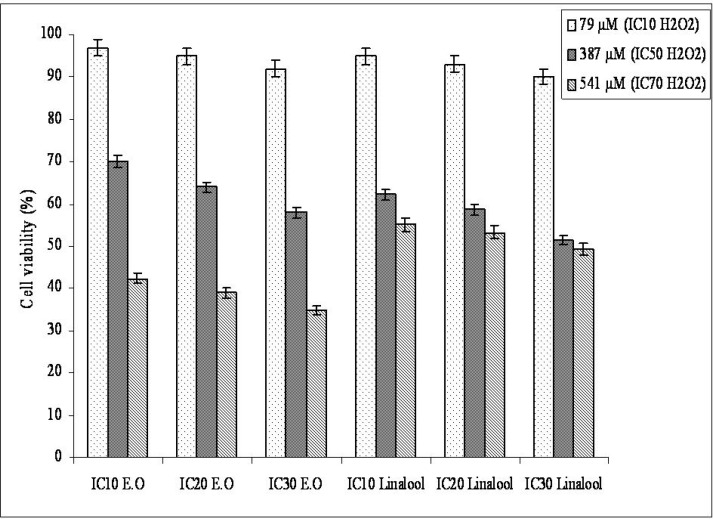
Protective effect of the essential oil from *O. majorana *and linalool aganist H_2_O_2_-induced cytotoxicity on Hep G2 cancer cells.

We examined the protective effect (antioxidant) of the essential oil and its oxygenated monoterpene component linalool against strong oxidant H_2_O_2_ on Hep G2 cells (Figure 1). Preincubation of cells with the essential oil and linalool increased cell viability against H_2_O_2_ cytotoxicity. These results indicate that the essential oil and linalool are capable of reducing H_2_O_2_-induced cytotoxicity. Essential oils from wild (camphor, sabinol/sabinyl acetate, thujene and eucalyptol (1,8 cineole) as the major compounds) and cultivated (camphor, eucalyptol (1,8 cineole), sabinol/sabinyl acetate, 3-oxo-beta ionone/isomethyl beta iononone as the major compounds) form of *Salvia pisidica *reduced the cytotoxicity induced by strong oxidant on H1299 and Hep G2 cells (37). Carvacrol and thymol were the components of *O. majorana*’s essential oil, protected parental, and epirubicin-resistant H1299 cells against H_2_O_2_-induced cytotoxicity, membrane, and DNA damage when the cells were preincubated with these two compounds at lower concentration (<IC_50_) before H_2_O_2_ incubation (38). Aqueous extract from *Morinda officinalis* showed protective effect to H_2_O_2_-induced cytotoxicity. Viabilities of cells exposed to 100 μmol H_2_O_2_ decreased below 50% and increased to a statistically significant extent up to 64.0% in the *Morinda officinalis*-treated group at 250 μg/mL (39).

E.O, Essential oil.

Values represent mean ± S.E. from three independent experiments.

The essential oil and linalool significantly decreased membrane-damaging on H_2_O_2 _treated Hep G2 cells (Table 3). The membrane-protective effect of linalool was found higher than essential oil. Preincubation with the IC_10_ and IC_20 _essential oil concentrations did not change the MDA amounts statistically according to incubation with IC_70_ H_2_O_2_. The similar results indicated that the essential oil from *O. onites, *carvacrol and, thymol showed membrane protective (antioxidant) effects at lower than IC_50_ concentrations on Hep G2 cells (18). In the future* O. majorana *and linalool’s protective effects against H_2_O_2 _will be good sources for hepatocellular carcinoma treatment which need more studies about understanding of the mechanism of the protective effects of essential oil and linalool against H_2_O_2_ on Hep G2.

**Table 3:** Amount of malondialdehyde in Hep G2 cells preincubated with *O. majorana* essential oil and its oxygenated monoterpene component linalool before H_2_O_2 _treatment

** Groups**	**MDA levels** ^f^ **(nmol/mg protein S.E.)**
IC_10 _E.O + IC_10_ H_2_O_2_	0.56 0.04 ab
IC_10 _E.O + IC_50_ H_2_O_2_	0.92 0.07 ab
IC_10 _E.O + IC_70_ H_2_O_2_	1.60 0.14 cd
IC_20 _E.O + IC_10_ H_2_O_2_	0.42 0.03 a
IC_20 _E.O + IC_50_ H_2_O_2_	0.75 0.07 ab
IC_20 _E.O + IC_70_ H_2_O_2_	1.52 0.66 cd
IC_30 _E.O + IC_10_ H_2_O_2_	0.38 0.06 a
IC_30 _E.O + IC_50_ H_2_O_2_	0.68 0.07 ab
IC_30 _E.O + IC_70_ H_2_O_2_	1.38 0.03 bc
IC_10 _Linalool + IC_10_ H_2_O_2_	0.42 0.08 a
IC_10 _Linalool + IC_50_ H_2_O_2_	0.78 0.07 ab
IC_10 _Linalool + IC_70_ H_2_O_2_	1.43 0.34 bc
IC_20 _Linalool + IC_10_ H_2_O_2_	0.35 0.06 a
IC_20 _Linalool + IC_50_ H_2_O_2_	0.72 0.07 ab
IC_20 _Linalool + IC_70_ H_2_O_2_	1.30 0.03 bc
IC_30 _Linalool + IC_10_ H_2_O_2_	0.29 0.03 a
IC_30 _Linalool + IC_50_ H_2_O_2_	0.65 0.07 ab
IC_30 _Linalool + IC_70_ H_2_O_2_	1.17 0.55 bc
IC_10_ H_2_O_2_	0.65 0.07 ab
IC_50_ H_2_O_2_	1.1 0.08 b
IC_70_ H_2_O_2_	1.70 0.34 cd
Control	0.23 0.11 a
0.5 % DMSO	0.22 0.20 a

MDA; malondialdehyde, E.O; essential oil, S.E.; Standart Error.

f Means (*n*=5) fallowed by different letters within column are significantly different (*p*≤ 0*.*05).

*Cytotoxicity of essential oil and linalool on Hep G2 cells*

**Figure 2:**
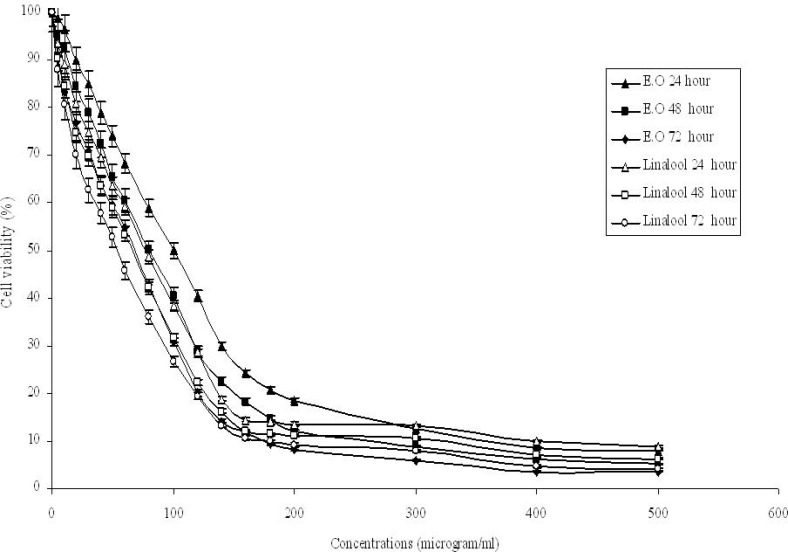
Cytotoxic activities of *O. majorana *essential oil and linalool for 24, 48 and 72 h on Hep G2 cells. Cell viability was assessed by CellTiter-Blue® Cell Viability Assay.

The effects of *O. majorana* essential oil and linalool on Hep G2 cells as assessed by CellTiter-Blue® Cell Viability Assay with different concentrations (5-500 μg/mL) are shown in Figure 2. Dose and time dependent inhibition by the essential oil and linalool were observed with IC_50_ values of 100, 80 and 63 μg/mL for essential oil and 81.5, 72.7 and 64.7 μg/mL at 24, 48 and 72 h, respectively. The IC_10_, IC_50 _and IC_70_ values of H_2_O_2_ incubations were 79, 387 and 541 μM respectively. DMSO (0.5%, v/v) did not affect the cell growth when treated for the same time periods. Linalool had more effective cytotoxic activity than essential oil on Hep G2 cells for 24 and 48 h incubations while essential oil had more effective cytotoxic activity than linalool for 72 h incubation. Those results showed that incubation time affected the essential oil’s and linalool’s cytotoxic effects on Hep G2 cells. *O. onites* essential oil and *O. majorana* essential oil’s second and main component respectively.In addition,carvacrol has been reported to be cytotoxic on Hep G2 cells and the essential oil was found to be less toxic than carvacrol and thymol for Hep G2 cells (18). The oils from the rhizome and the aerial part of *A*. *mollissima *showed cytotoxicity on Hep G2 cells (40). *T. revolutus* Célak essential oil and its two main components (cymene and γ-terpinene) were found cytotoxic in concentration- and time-dependent manners in Hep G2 cells (41). Plant extracts from *O. majorana* showed cytotoxicity and anti-proliferative effect on Jurkat cells (42). Methanolic extracts of some medicinal plants showed cytotoxic effects on A549, MCF-7, Hep G2 and HT-29 cells (48).

**Figure 3:**
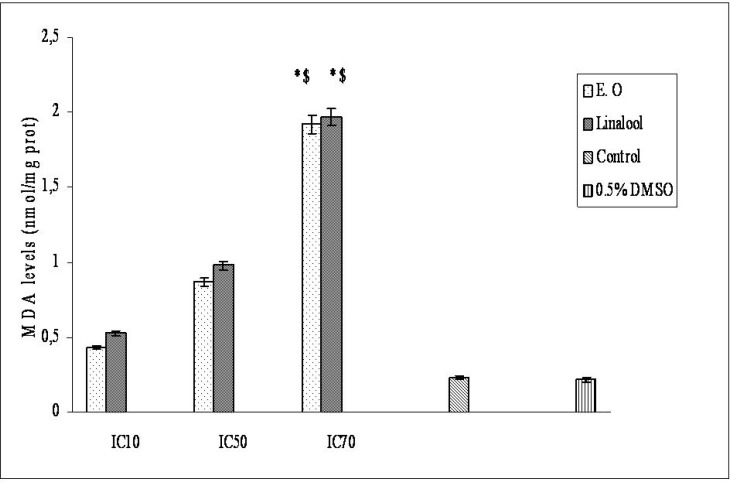
Dose-dependent membrane-damaging effects of essential oil from *O. majorana *and linalool on Hep G2 cells.

*O. majorana* essential oil and linalool induced membrane damage on Hep G2 cells are shown in Figure 3. Membrane-damaging effects of the essential oil and linalool increased with accelerating concentrations. Linalool membrane-damaging effect was found stronger than essential oil membrane-damaging effect. In one of our previous study linalool had more effective membrane damaging effect than *O. majorana* essential oil on epirubicin-resistant H1299 cells but on parental cells, the essential oil had more effective membrane damaging effect than linalool (43). Biochemical changes like membrane structure in drug resistant cells will affect the essential oil’s and linalool’s cytotoxicity. Differences between the essential oil’s and linalool’s chemical structures (as a result of they will produce different ROS and amounts) will be the reason for having different cytotoxic effects on Hep G2 cells of the essential oil and linalool. 

E.O, Essential oil.

Values represent mean ± S.E. from three independent experiments.

* Significantly different from control (untreated cells) and 0.5% (v/v) DMSO control; ^$^ Significantly different from IC_50 _essential oil and linalool treatment in Hep G2 cells cells.

Synergistic functions of the various molecules contained in an essential oil, in comparison to the action of one or two main components of the oil, seems questionable. However, it is possible that the activity of the main components is modulated by other minor molecules (44-46). Moreover, it is likely that several components of the essential oil play a role in defining the fragrance, density, texture, colour and above all, cell penetration (47), lipophilic or hydrophilic attraction as well as fixation on cell walls membranes, and cellular distribution. The last feature is very important because the distribution of the oil in the cell determines the different types of radical reactions produced, depending on their compartmentation in the cell.

*O. majorana* essential oil and its oxygenated monoterpene component linalool showed antioxidant and cytotoxic activity depending on concentrations and time manner. Those results indicate that concentrations are important in their usage. *O. majorana*’s and linalool’s antitumoral properties suggest that they could be the potential source of hepatocellular carcinoma treatment.
